# Our Annual Volume of Transactions

**Published:** 1883-03

**Authors:** 


### OUR ANNUAL VOLUME OF TRANSACTIONS.

Month after month has passed away since the last meeting of the State Medical Association, until we are now within a few weeks of another annual reunion, and yet the published records of the last session have not been distributed to the members of that body. There may be a sufficient reason for this unfortunate delay, but no explanation has been made to those who are directly interested, and who have a right to know why the usages and necessities of the Association have been thus apparently disregarded.

The last meeting, held in this city, was considered one of the most successful in the history of the organization, and it was hoped that new life and increased vigor would be infused into the Association, which for several years had seemed to languish; but whatever of enthusiasm was kindled at that time, we fear has suffered a disastrous repression by the practical burial for months, it may be forever, of the proceedings of the session.

It is to be hoped, for its own protection, that the Association will take the necessary action to prevent in future the occurrence of such a calamity—for the withholding of the transactions from its members and from the public for nearly a year cannot be regarded otherwise than as a serious misfortune to the body.

